# Case of Obstruction of the Bowels, with Remarks

**Published:** 1784

**Authors:** 


VI. *Caſe of Obſtruction of the Bowels, with Remarks. Communicated in a letter to Dr.* Simmons, *by* Robert Willan, M. D. *Phyſician to the*

*the Finsbury Dispensary, and to the Public Dispensary in Carey-street.*

A LADY, fifty-two years old, of an irritable habit, subject to frequent attacks of pain in her bowels, and generally costive, was seized in the evening of April 2, 1784, with the usual symptoms of colic, attended with almost incessant vomiting.—She had had a slight evacuation by stool the preceding morning.

A purging mixture, with *Sal. Glauber.* and *Pulv. Jalap.* was first prescribed, and properly retained, but without producing the desired effect. She continued very restless and uneasy all that night. The vomiting also returned, but was stopped in the morning, by an anodyne draught, which contained forty drops of *Tinct. Theb.*

In the course of the next day, she had clysters of assafœtida and *Sal. Cathartic.* and in the evening took a scruple of *Extract. Cathartic.* ten grains of calomel, and a grain of *Extract Thebaic.* made into pills.

April 4. No alteration had taken place in her complaint. The pain returned in very severe paroxysms, with some intervals of ease. About

About four ounces of blood were drawn from the abdomen, by cupping; and a large bliſter applied. Oily clyſters, with aſſafœtida, were alſo given, and the pills continued.

5th. No good effect yet appeared from the above applications. She remained in the ſame ſtate for ſome days longer, during which ſhe took different purgative mixtures; with *Reſin. Jalap. Ol. Ricin,* &c. and *Extr. Thebaic.* occaſionally. The warm bath was alſo employed repeatedly, and a tobacco clyſter adminiſtered. She complained of a diſagreeable taſte in her mouth from the tobacco, as ſhe had before done of the aſſafœtida. Still, however, our hopes were diſappointed, no evacuation being produced. It is ſingular, that in a diſorder of this kind, which had now continued ſix days, there ſhould have been no heat or fever, no hardneſs or mark of irritation in the pulſe; the patient herſelf, in the intervals of eaſe from excruciating pain, being always cool, collected, and ſenſible.

During the laſt three days, ſhe had had frequent hiccup, but that ſymptom went off again.

As it ſeemed in vain to purſue the purgative plan any farther, the application of cold water, as recommended by many eminent phyſicians, was the meaſure next adopted. It was judged, however, more convenient to apply it in the form of ice, properly guarded. This being done accordingly, April the 8th, ſhe bore the application for thirty-five minutes, though the pain occaſioned by it was exceſſive, more eſpecially ſo from the recent ſoreneſs of the bliſter. It excited much commotion in her bowels, and borborygmus, yet no evacuation of fœces or wind followed. We found her the next day in her uſual ſtate, but low, and unwilling to try the effects of medicine any farther.

She had been ſeen thus far, at different times, by Drs. W. Saunders, Lettſom, and Grant: the two latter now diſcontinued their attendance, thinking the caſe hopeleſs. Dr. Saunders and myſelf agreed to viſit her occaſionally, and watch any opportunity that might offer for relief. She was directed in the mean time to take light nouriſhing food, with a little wine; and had anodyne emollient clyſters injected twice a day.

April 10. In the morning ſhe broke wind downwards once or twice, and ſeemed on the whole eaſier and better. Finding circumſtances thus favourable, and being importuned by her anxious anxious friends to attempt ſomething more, we gave directions to make trial of quickſilver.—She took it in doſes of two drachms, to the amount of ſix ounces; but from the ſenſe of weight and uneaſineſs it occaſioned, ſhe could not be prevailed on to take more of it. Copious watery injections were likewiſe thrown up to ſecond its effect.

On the 12th, a few ſmall globules of quickſilver appeared in a clyſter, which had been retained four hours. This encouraged us again to preſcribe a purgative mixture of *Infuſ. Sennæ* & *Pulv. Scammon.* which being diſagreeable to her ſtomach, was exchanged for the *Pil. e Colocynth. cum Aloe*, & Calomel.

On the 17th and 18th, there ſeemed to be a ſmall quantity of fœculent matter brought off in the clyſters. There was alſo ſeveral times a conſiderable mucous diſcharge, which came off ſpontaneouſly. From this time, though the ſame plan was perſiſted in, no evacuation could be produced, nor did any more of the quickſilver appear.

She now refuſed to make farther trial of medicines; and wiſhed to reſign herſelf to her fate. The pains continued to return at frequent and irregular intervals: her pulſe was often very low and and intermitting, but generally better every other day. Once or twice ſhe found herſelf ſo well, that ſhe got up, and, with a little aſſiſtance, took ſeveral turns round the room.—At the requeſt of her friends, who were unwilling to omit any poſſible means of relief, ſhe ſubmitted to be electrified; and bore with great firmneſs ſeveral ſmart ſhocks, paſſed through the abdomen in different directions. This was not, however, attended with any particular effect.

April 23d. Her abdomen was conſiderably diminiſhed in ſize, and leſs tenſe. We had before frequently obſerved a variety in this reſpect, though without any diſcharge of flatus either way.

She continued much in the ſame ſtate till the 1ſt of May; when, after a very reſtleſs night, ſhe was ſeized with moſt excruciating fits of pain, and died about ſix o'clock in the morning.

She had herſelf requeſted that her body ſhould be opened and inſpected after death; which was accordingly performed the day following, by her Surgeon, Mr. Lowdell, of Queen-ſtreet, Southwark. We found the bowels amazingly diſtended throughout, particularly the *caput cæcum coli*, and containing, on the whole, not lefs than four gallons of feculent matter, in a fluid ftate. The whole tract of the inteftines was inflamed, in many places fphacelated, and too tender to bear the flighteft handling.

The conftriction was found to be at the lower part of the figmoid flexure of the colon, near the top of the os facrum, and at the beginning of the rectum. For about the length of an inch, the inteftine was contracted, fo that nothing could pafs. The ftricture had probably formed very gradually, this portion of the bowels being quite hard and callous. Some quickfilver was found above the ftricture; a part of it feemed alfo to have been triturated with the mucus of the bowels into a black gelatinous mafs. The other abdominal vifcera were in a natural ftate. One circumftance, however, deferves to be particularly remarked, which is, that we obferved feveral tubercles of confiderable fize adhering to the fundus uteri on the outfide, which, upon examination, were found to be very hard, and of an offeous nature.

### *Obſervations and Deductions.*

1ſt, IT appears from this caſe, that extenſive inflammation, and even mortification, may take place from a gradual diſtention of the bowels, without producing heat, fever, or any of the common inflammatory ſymptoms.

2dly, The time during which it is poſſible to ſubſiſt under ſuch a diſorder, without any alvine diſcharge, merits attention. This patient remained without any evacuation by ſtool, upwards of thirty days; whereas in ſimilar caſes, recorded by Morgagni and Lieutaud, we find that the patients ſeldom ſurvived above ten or twelve days.—She made water freely all the time, and in proper quantity.—The vomitings which occaſionally occurred, ſeemed only to eaſe the ſtomach of a temporary load, no feculent matter ever appearing in the fluids diſcharged.

3dly, The different ſize, and degree of tenſion, of the abdomen, without any evident cauſe, are not ſo eaſy to be explained. Perhaps theſe might be owing merely to a change of place in the flatus, to its being more or leſs diffuſed through the bowels at different times.

4thly,

4thly, The hard bony ſubſtances found on the uterus might probably occaſion firſt a ſpaſm or contraction of the inteſtine, and afterwards, by their repeated irritation, that callous ſtate of the coats which appeared on diſſection. With reſpect to the origin of theſe tubercles, little judgement could be formed. We only learnt that her bowel complaints came on, and her general health was much impaired, in conſequence of a ſevere labour ſeveral years before, and the delivery of a dead child, by the uſe of inſtruments.

5thly, Quickſilver, taken into the bowels in conſiderable quantity, though retained for ſome time, does not always produce dangerous effects from its bulk or momentum. It is triturated up with the mucus of the bowels, by their conſtant periſtaltic motion, and ſoon loſes its proper form.

6thly, Conſtrictions in the larger inteſtines are not attended with the ſame acute and violent ſymptoms, viz. of fever, ſharp pain, perpetual vomiting, hiccup, great proſtration of ſtrength, &c. which generally ſoon prove fatal in contractions or ſpaſms of the ſmaller bowels. This obſervation may contribute towards aſcertaining more nearly the place of ſuch obſtruc-

 tions.

tions. They are not fo ufual in the larger inteftines. Lieutaud has collected fome inftances of them in the colon and rectum, and alfo of large tumors filling up the whole cavity whereby the paffage of fæculent matter was prevented. In all thefe cafes, the fymptoms were not very urgent, and the patients fubfifted under the diforder from feven to eighteen days, and upwards; whereas the coalefcence, or conftrictions of the fmall inteftines, mentioned by him, proved fatal in two, three, or four days; which is conformable to our common experience.

7thly, When, from this diagnoftic, or other circumftances, the obftacle is concluded to be fomewhere in the great inteftines, if active purgatives and ftimulant enemas fail on their firft application, we fhould be cautious of perfifting in the ufe of fuch irritating remedies, which often give unneceffary pain, and rather tend to aggravate the diforder. The next proper meafure is to examine the ftate of the large inteftines, fo far as it can be done conveniently, by means of a candle, or bougie of a proper fize; and thence to form our judgement more exactly concerning the feat of the diforder. If it be within reach, fuitable means muft be made ufe of, according to the nature of the cafe, as whether ther it be from contraction of the coats, hard tumors, or indurated excrement. There will be much nicety and difficulty with respect to the use of force, and the direction in which it should be applied, if dilatation be thought necessary. From the situation of the contraction in the present case, and the state of the intestinal coats, it would have been impracticable. It was, indeed, proposed in consultation, to try some experiments of this kind, but we were prevented by the scruples of the patient, and the circumstance of copious watery injections being easily retained for some hours; whence it might be concluded, that the seat of the disorder was out of our reach.

Tumors in the rectum, which by their size block up the cavity of the intestine, also admit but of little relief. I lately met with an unfortunate case of this kind in Mrs. Wetherell, of St. John-street, Smithfield, who had been for some time liable to obstinate costiveness, and stoppage of urine.

On the 7th of October last, having been five days without a stool, she took half a drachm of Extract. Cathartic. and four grains of Calomel: and the day following, an ounce and a half of

Caſtor Oil, in divided doſes, without any effect. An examination was then made, according to the plan above mentioned, by Mr. Andree, Surgeon, who found a hard reſiſting tumor about five inches from the verge of the anus. Having paſſed this with much difficulty, another ſwelling preſented itſelf, of equal ſize and firmneſs with the former, and created an obſtacle totally impaſſable by any inſtrument he could make uſe of. The patient languiſhed in great miſery about ſeven days longer. Nothing preternatural was diſcovered in the bladder, by uſing the catheter. However, we were at no loſs in accounting for the nature of theſe ſwellings, as ſhe had been previouſly ſubject to hæmorrhoidal affections.

